# ROS Modulating Effects of Lingonberry (*Vaccinium vitis-idaea* L.) Polyphenols on Obese Adipocyte Hypertrophy and Vascular Endothelial Dysfunction

**DOI:** 10.3390/nu13030885

**Published:** 2021-03-09

**Authors:** Katarzyna Kowalska, Radosław Dembczyński, Agata Gołąbek, Mariola Olkowicz, Anna Olejnik

**Affiliations:** 1 Department of Biotechnology and Food Microbiology, Poznan University of Life Sciences, 48 Wojska Polskiego St., 60-627 Poznan, Poland; katarzyna.kowalska@up.poznan.pl (K.K.); radoslaw.dembczynski@up.poznan.pl (R.D.); agata.golabek@up.poznan.pl (A.G.); 2 Jagiellonian Centre for Experimental Therapeutics, Jagiellonian University, 14 Bobrzynskiego St., 30-348 Krakow, Poland; mariola.olkowicz@jcet.eu

**Keywords:** polyphenols, anthocyanins, lingonberry, antioxidant potential, anti-obesity, anti-inflammatory, 3T3-L1 adipocytes, hypertrophy, adipokines, endothelial dysfunction

## Abstract

Oxidative stress and dysregulated adipocytokine secretion accompanying hypertrophied adipose tissue induce chronic inflammation, which leads to vascular endothelial dysfunction. The present study investigated the ability of anthocyanin (ACN) and non-anthocyanin polyphenol (PP) fractions from lingonberry fruit to mitigate adipose tissue hypertrophy and endothelial dysfunction using 3T3-L1 adipocytes and human umbilical vein endothelial cells (HUVECs). This study showed that the PP fraction decreased intracellular ROS generation in hypertrophied adipocytes by enhancing antioxidant enzyme expression (*SOD2*) and inhibiting oxidant enzyme expression (*NOX4*, *iNOS*). Moreover, PP and ACN fractions reduced triglyceride content in adipocytes accompanied by downregulation of the expression of lipogenic genes such as *aP2*, *FAS*, and *DAGT1*. Treatment with both fractions modulated the mRNA expression and protein secretion of key adipokines in hypertrophied adipocytes. Expression and secretion of leptin and adiponectin were, respectively, down- and upregulated. Furthermore, PP and ACN fractions alleviated the inflammatory response in TNF-α-induced HUVECs by inhibiting the expression of pro-inflammatory genes (*IL-6*, *IL-1β*) and adhesion molecules (*VCAM-1*, *ICAM-1*, *SELE*). The obtained results suggest that consuming polyphenol-rich lingonberry fruit may help prevent and treat obesity and endothelial dysfunction due to their antioxidant and anti-inflammatory actions.

## 1. Introduction

Obesity is an independent risk factor for cardiovascular disease and one of the leading causes of the increased risk of dyslipidemia, insulin resistance, hypertension, and atherosclerosis both in adults and children [1]. In obesity, white adipose tissue (WAT) by excessive fat accumulation in hypertrophied adipocytes becomes dysfunctional, which leads to chronic inflammation, oxidative stress, and dysregulated adipokine secretion that contributes to type 2 diabetes mellitus and is also independently associated with coronary endothelial dysfunction [2,3]. Hypertrophic adipocytes are the essential factor linking positive energy balance, diabetes, and cardiometabolic diseases [2]. WAT acts as an endocrine organ and via secreted adipokines and cytokines mediates cross-talk between visceral or subcutaneous WAT and cardiovascular tissues. Adipokines such as leptin, adiponectin and resistin, cytokines, TNF-α, IL-1β, IL-6, IL-8, and MCP-1, and reactive oxygen and nitrogen species (ROS and RNS) affect endothelial dysfunction development through direct and indirect mechanisms [4]. In addition, perivascular adipose tissue (PVAT), mainly from obese individuals, promotes local inflammation and endothelial function impairment. PVAT contributes to vascular homeostasis by producing vasoactive compounds such as adipokines, ROS, and nitric oxide (^•^NO). By secreting a wide range of bioactive molecules, PVAT influences vascular smooth muscle cell contraction, proliferation, and migration [4].

The endothelial cells that line the vasculature’s inner wall regulate homeostatic functions, and their dysfunction is an early predictor of atherosclerosis and cardiovascular diseases [5]. Oxidative stress contributes to endothelial cell activation, priming it for adhesion, infiltration, and immune cell activation, leading to a low-grade inflammatory phenotype in the vasculature [5,6]. ROS can alter endothelium-dependent vascular relaxation via enhanced degradation of NO [6]. Endothelial dysfunction can be reversed, which might delay or even prevent the progression of atherosclerosis and improve arterial function and reduce the incidence of cardiovascular events [5]. Recent clinical studies have demonstrated that non-pharmacological and pharmacological therapies targeting obesity and insulin resistance ameliorate endothelial function and reduce low-grade inflammation [7]. These findings have shown the association of obesity, insulin resistance, and endothelial dysfunction; therefore, reducing pathological adipocyte function in obesity should be the goal in cardiovascular disease prevention. Therapeutic and nutritional strategies that decrease oxidative stress and inflammation in hypertrophied adipose tissue may become a key target to prevent cardiovascular disease [7].

Berries are rich sources of polyphenols, such as flavonols, phenolic acids, and anthocyanins, and epidemiological studies have reported an association between an increase in berry fruit intake with a decrease in obesity and cardiovascular disease [8]. Berry fruits are known as natural antioxidants, and due to their high antioxidant potential, they are increasingly often referred to as natural functional foods [9]. Lingonberries are classed as “superfruits”, being particularly rich in antioxidants such as vitamins C, A, and E (tocopherol) and polyphenols [10]. In vitro and in vivo studies have indicated various health beneficial effects of lingonberries such as anti-inflammatory [11], antioxidant [11], and antiproliferative activities [8,9]. Moreover, lingonberries have been shown to prevent diet-induced obesity and low-grade inflammation in diabetic animals [12]. Our previous study showed the anti-inflammatory potential of aqueous extract of freeze-drying lingonberry fruit [11]. The extract regulated pro-inflammatory (IL-6, MCP-1, and IL-1β) and anti-inflammatory (IL-10) gene expression in inflamed TNF-α-induced 3T3-L1 adipocytes and suppressed the inflammatory response in activated RAW 264.7 macrophages by downregulating expression of proinflammatory mediators (TNF-α, IL-1β, IL-6, MCP-1, iNOS, COX-2). In addition, significant antioxidant effects were observed in inflamed adipocytes treated with the lingonberry fruit extract. The intracellular ROS accumulation decreased as a result of enhanced expression of antioxidant defense enzymes (SOD, catalase, GPx) and inhibited pro-oxidant enzyme (NADPH oxidase 4) [11].

The present study investigated the lingonberry fruit anthocyanin (ACN) fraction and non-anthocyanin polyphenol (PP) fraction ability to prevent and treat hypertrophic obesity and endothelial dysfunction imitated in the in vitro models. The effect of ACN and PP fractions on the molecular pathways in oxidative stress, inflammation, and dysregulated adipokine secretion was analyzed in obese hypertrophied 3T3-L1 adipocytes. Protective potential against endothelial dysfunction was evaluated using TNF-α-induced human umbilical vein endothelial cells (HUVECs).

## 2. Materials and Methods

### 2.1. Preparation of Anthocyanin and Non-Anthocyanin Polyphenol Fractions

The frozen lingonberry (*Vaccinium vitis-idaea* L.) fruit obtained from the DANEX company (PHU “DANEX”, Wieleń, Poland) were homogenized to fruit pulp, which was subsequently frozen at −80 °C and subjected to freeze-drying according to the procedure described previously [11]. The fruit powder was suspended in a water solution of 0.75% (v/v) acetic acid. The proportion of solids (g) and the extractant (mL) was 1:10. After mixing in a vortex mixer for 30 s, the suspension was placed in a sonic bath (5 min, 20 °C). Again, the extraction mixture was stirred in a vortex mixer for 30 s and left to stand at 20 °C. After 10 min, the sample was centrifuged at 3600× *g* (10 min, 20 °C), and the obtained supernatant was collected. The fresh extractant was poured into the remaining solids to start the second extraction stage. The procedure of the second stage was the same as the first one. The extracts of both stages were combined and centrifuged at 12,000× *g* to remove tiny fruit residues.

In the next step of fraction preparation, removal of sugars and organic acids from the extract was performed. The separation was carried out with AKTA Explorer 100 Air (GE Healthcare, Chicago, IL, USA) chromatography system equipped with an XK 26/20 glass column (GE Healthcare, Chicago, IL, USA). The column was filled with 40 mL of Amberlite XAD-7 HP macroporous adsorbent resin (DuPont, Wilmington, DE, USA). Before injection to the column, the solution (50 mL of the extract obtained in the extraction stage) was filtered using a 0.45-µm pore size syringe filter (Millex-HV Durapore^®^ PVDF) membrane with glass fiber prefilter (Merck Millipore, Burlington, MA, USA). Three eluents were applied: A—5% (*v/v*) formic acid, B—methanol, C—0.1% (*v/v*) formic acid. The solutions of formic acid were prepared by mixing an appropriate amount of formic acid with deionized water. The eluent flow rate was adjusted at 5 mL/min. During separation, the following chromatographic program was employed: Column equilibration: 95% A, 5% B, 3 CV (column volume); sample injection‒50 mL of the extract; washing unbound substances-1: 100% C, 6 CV; washing unbound substances-2: 100% A, 1 CV; elution: 20% A, 80% B, 5 CV; column wash: 100% B, 2.5 CV.

The whole effluent of the elution stage which showed the absorbance (monitored at λ = 280, 320, and 520 nm) was evaporated to dryness at 30 °C using a rotary evaporator (Laborota 4003 HB control, Heidolph, Germany). The solids were dissolved in a water solution of 0.75% (*v/v*) acetic acid. The solution was transferred to glass vials and frozen at −85 °C, and then placed in a freeze dryer Beta 1-16 (Martin Christ, Germany). Freeze-drying was carried out for 48 h. The actual drying took place under the pressure of 10 Pa for 40 h (20 h at a shelf temperature of −15 °C and 20 h at 15 °C). The final drying was performed at a temperature of 22 °C for 8 h without pressure control. Solid preparations were stored in hermetically sealed vials under the nitrogen atmosphere at −85 °C.

The vial solid content was dissolved in the water solution of 5% (*v/v*) formic acid and filtered using a 0.45-μm pore size filter (Merck Millipore). The separation of anthocyanins from the other polyphenol compounds in the samples was performed using an ÄKTA Explorer 100 Air chromatograph, equipped with a UV/VIS detector and an Agilent Zorbax SB C18 column (250 × 21.2 mm). The separation was carried out at 20 °C. The flow rate of the liquid phase was 21 mL/min. Two eluents were applied: A‒5% (*v/v*) formic acid in water and B‒methanol. After the column equilibration (95% A, 5% B, 3 CV) and sample injection in the volume of 2 mL, the separation was performed in a complex gradient. The gradient program was as follows: 5% B‒0.5 CV; 20% B‒2 CV; 20% B‒1.2 CV; 30% B‒3.5 CV; 30% B‒1.2 CV; 45% B‒3.5 CV; 45% B‒1.2 CV; 100% B‒2.5 CV; 100% B‒2.5 CV. The effluent with absorbance at λ = 520 nm was collected and denoted as an anthocyanin (ACN) fraction. The outflow showing absorbance at λ = 320 nm was also gathered and denoted as the non-anthocyanin polyphenol fraction (PP). Both fractions were evaporated, dissolved in a water solution of acetic acid, freeze-dried, and stored as described above. All chemicals used to prepare the ACN and PP fractions were purchased from Sigma–Aldrich (Merck Group, Poznań, Poland).

### 2.2. Polyphenol Identification and Quantification in ACN and PP Fractions

Polyphenol composition of ACN and PP fractions was analyzed by the HPLC-DAD-ESI-MS method on an Agilent 1200 series HPLC system (Agilent Technologies, Inc., Santa Clara, CA, USA) equipped with a G1315D photodiode array detector and coupled online with an Agilent 6224 time-of-flight MS system. Chromatographic separations were carried out on a 150 × 2.1 mm, 3-μm C18 column (Advanced Chromatography Technologies, Aberdeen, Scotland). A previously published study details the separation conditions (mobile phase, gradient elution program, flow rate, sample injection volume) [11]. The HPLC chromatograms were recorded at 280, 325, 355, and 520 nm, recommended to detect flavan-3-ols, hydroxycinnamic acid derivatives, flavonols, and anthocyanins, respectively.

Polyphenol compounds in ACN and PP fractions were quantified as equivalents of cyanidin-3-*O*-glucoside (anthocyanins), catechin ((epi)catechin and procyanidins), 4-hydroxybenzoic acid (hydroxybenzoic acid derivatives), ferulic acid (ferulic acid derivative), chlorogenic acid (3-*O*-caffeoylquinic acid), *p*-coumaric acid (coumaric acid derivative), trihydroxybenzoic acid‒gallic acid (benzoic acid and arbutin derivatives), and quercetin (quercetin glycosides). All samples were injected in triplicate from independently prepared solutions of ACN and PP fractions.

After passing through the DAD detector, column eluate was directed to the MS system fitted with an electrospray ionization (ESI) source operated in positive ion and negative ion mode. A previously published article presents ESI-MS parameters employed for identifying phenolic compounds in ACN and PP fractions [11]. Instrument control, data collection, and analysis were achieved with MassHunter B.04.00 software (Agilent Technologies, Inc., Santa Clara, CA, USA). Sigma–Aldrich supplied phenolic standards and other reagents for HPLC/DAD/MS analysis.

### 2.3. 3T3-L1 Adipocyte Culture and Treatment

Mouse preadipocyte 3T3-L1 cells were obtained from the American Type Culture Collection (ATCC, CL-173). The cells were cultured at 37 °C under a 5% CO_2_ atmosphere in Dulbecco’s modified eagle’s medium (DMEM) (Sigma–Aldrich, Poznań, Poland) with 10% (*v/v*) fetal bovine serum (FBS) (Gibco, Thermo Fisher Scientific Polska, Warsaw, Poland) supplementation. 3T3-L1 preadipocytes were subjected to the differentiation process following the protocol described previously [11]. Preadipocytes were seeded at a density of 2.5 × 10^4^ cells/cm^2^ into 12-well plates and cultured until they reached confluence. Then they were stimulated for 2 days by a differentiation mixture which contained 0.25 µM of dexamethasone (Sigma–Aldrich, Poznań, Poland), 0.5 mM 3-isobutyl-1-methylxanthine (Sigma–Aldrich, Poznań, Poland) and 1 µM of insulin (Sigma–Aldrich, Poznań, Poland) in DMEM with 10% FBS. The medium was replaced with DMEM supplemented with 10% FBS and 1 µM insulin. After 2 days, the culture medium was replaced with DMEM with 10% FBS addition and refreshed at 2-day intervals until analysis on day 12. 3T3-L1 adipocytes were treated for 24 h with ACN and PP fractions at concentrations of 5, 10, and 20 µg/mL.

### 2.4. HUVEC Culture and Treatment

Human umbilical vein endothelial cells (HUVECs) were obtained from ATCC (CRL-1730). HUVECs were cultivated in F-12K medium (ATCC) supplemented with 10% FBS (Gibco), endothelial cell growth supplement from bovine neural tissue (30 µg/mL) (Sigma–Aldrich, Poznań, Poland), and heparin (100 µg/mL) (Sigma–Aldrich, Poznań, Poland). HUVECs were seeded at a density of 6 × 10^3^ cells/cm^2^ onto 24-well plates coated with rat tail collagen solution (Sigma–Aldrich, Poznań, Poland). Then 24-h cultures of HUVECs were exposed for 3 h to ACN and PP fractions at the concentrations of 0.1, 1, and 10 µg/mL and subsequently treated with TNF-α (10 ng/mL) (Sigma–Aldrich, Poznań, Poland) for an additional 3 h to induce inflammation.

### 2.5. Cell Viability Assay

The viability of hypertrophied 3T3-L1 adipocytes and TNF-α-induced HUVECs, non-treated and treated with ACN and PP fractions, were analyzed applying the MTT (3-(4,5-dimethylthiazol-2-yl)-2,5-diphenyltetrazolium bromide) assay (Sigma–Aldrich, Poznań, Poland) following the procedure described previously [13]. Low concentrations of ACN and PP fractions applied for cell treatment did not affect the color of medium and absorbance reading in the MTT test.

### 2.6. Determination of Intracellular ROS Production

ROS generation in 3T3-L1 adipocytes was measured using the nitro blue tetrazolium (NBT) assay based on the procedure described previously [14]. After 90-min incubation in 0.2% NBT (Sigma–Aldrich, Poznań, Poland) solution, cells were washed with phosphate-buffered saline and fixed with methanol. After extraction of the formazan using KOH and DMSO, absorbance was read at 620 nm (Tecan Infinite M200, Tecan Group Ltd., Männedorf, Switzerland).

### 2.7. Measurement of Intracellular Lipid Content

The effect of PP and ACN fractions on lipid content in hypertrophied adipocytes was determined by the Oil Red O (Sigma–Aldrich, Poznań, Poland) staining method described previously [13] and by total triglycerides (TG) measurement using the Adipogenesis Assay Kit (Sigma–Aldrich, Poznań, Poland) in accordance with the manufacturer’s instruction. Intracellular TG content was determined by an enzyme assay. A colorimetric product corresponding to the TG present was measured at 570 nm. TG concentration was calculated based on the curve plotted for the TG standard.

### 2.8. RNA Extraction and Real-Time PCR Analysis

3T3-L1 adipocytes and HUVECs were treated with TRI-Reagent (Sigma–Aldrich, Poznań, Poland) for total RNA isolation. First-strand cDNA synthesis was performed with 1 µg of total RNA using a Transcriptor First Strand cDNA Synthesis Kit (Roche Diagnostics, Poland) based on the manufacturer’s instruction. Gene expression quantification was conducted using a real-time PCR system (SmartCycler DX real-time PCR System Cepheid, Sunnyvale, CA, USA). PCR mixture in a final volume of 25 µL included a cDNA sample (1 µL), specific forward and reverse primers (5 µM/1 µL), and SYBR^®^ Select Master Mix (12.5 µL) (Life Technologies, Carlsbad, CA, USA). Primer sequences are shown in Table S1. The PCR cycling conditions included an initial denaturation at 94 °C for 10 min, followed by 40 PCR cycles: 40 s at 95 °C, 30 s at 59 °C, and 30 s at 72 °C. The relative gene expression was calculated using the 2^−∆∆CT^ method. Transcript levels were normalized to β-actin for 3T3-L1 adipocytes and GAPDH for HUVECs. Relative mRNA expression was expressed as fold change compared with control (untreated) cells. All reactions were performed in triplicate.

### 2.9. Determination of Adipokine Production

Leptin and adiponectin concentrations were measured with ELISA kits (Sigma–Aldrich, Poznań, Poland) following the manufacturer’s protocols. Quantitation was performed using the calibration of standards. Each standard and sample was assayed in triplicate. Inter-assay and intra-assay coefficients of variability were calculated respectively at 12.5% and 9.3% for leptin and 11.2% and 7.9% for adiponectin.

### 2.10. Statistical Analysis

Statistical analysis was performed using the STATISTICA version 13.3 software (Statsoft, Inc., Tulsa, OK, USA). One-way analysis of variance (ANOVA) and Tukey’s post hoc test were applied to estimate the differences between multiple groups’ mean values. Levene’s test verified the equality of variances assumption. Statistical significance was set at *p* < 0.05.

## 3. Results and Discussion

### 3.1. Polyphenol Composition in the Lingonberry ACN and PP Fractions

The study focused on two polyphenolic preparations separated from lingonberry fruit: the anthocyanin ACN fraction and non-anthocyanin PP fraction. The polyphenol profiles in ACN and PP fractions determined based on HPLC-DAD-ESI-MS analysis are presented in Table 1. ACN fraction consisted of three main anthocyanin compounds, contained in lingonberry fruit extract [11], which are cyanidin-based derivatives, including 3-*O*-galactoside (82.5%), 3-*O*-arabinoside (13.0%), and 3-*O*-glucoside (4.5%) (Table 1A).

The purity of ACN preparation was evaluated at 97.3%; among the non-anthocyanin constituents, 1-*O*-Benzoyl-*β*-glucose was identified by HPLC-ESI-MS analysis in positive ion mode (precursor ion at m/z 307.079, product ion at m/z 185.0432). The PP fraction contained polyphenolic compounds belonging to three predominant groups: Flavan-3-ols, hydroxycinnamic acid derivatives, and flavonols, which accounted for 40.4%, 22.8%, and 31.0%, respectively. In addition, the anthocyanin compounds’ residue (5.8%) was detected in the PP fraction with cyanidin-3-*O*-galactoside as dominant anthocyanin, cyanidin-pentoside, and cyanidin 3-*O*-(6’’-acetyl)-glucoside (Table 1B), trace amounts of which have been identified previously in the original lingonberry fruit extract [11]. In the PP fraction, the following polyphenols were quantified in a significant amount (>5%): A- and B-type procyanidins, catechin, 3-*O*-caffeoylquinic acid, ferulic acid−hexoside, quercetin and its derivatives (3-*O*-galactoside, 3-*O*-arabinofuranoside, 3-*O*-rhamnoside). Table 1B shows mass spectral data of all polyphenolic compounds tentatively identified in the PP fraction of lingonberry fruit.

### 3.2. The Effect of PP and ACN Fractions on ROS Generation in Hypertrophied 3T3-L1 Adipocytes

**Figure 1:**
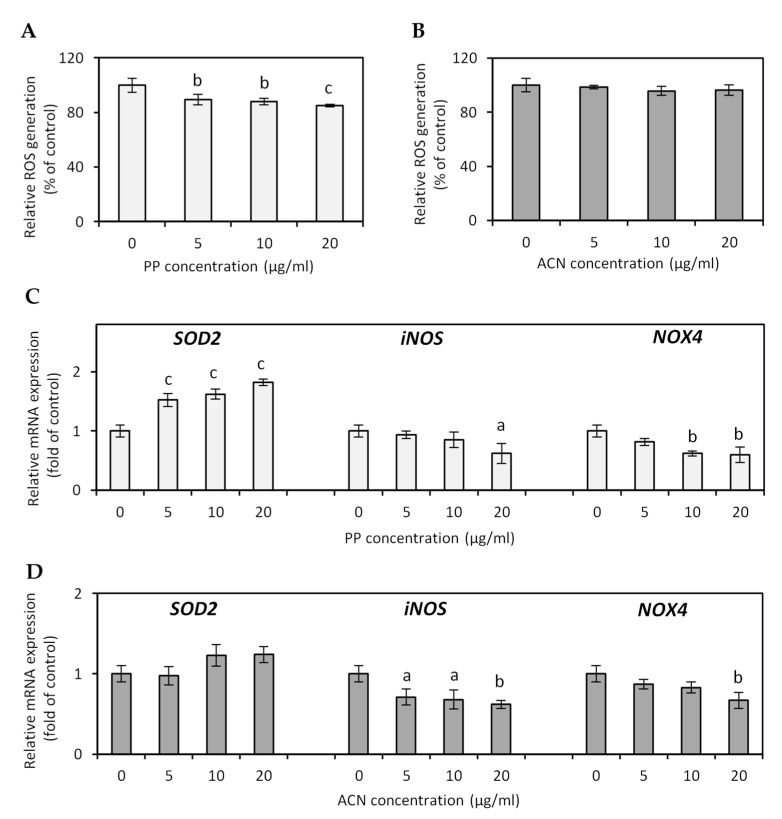
Effect of anthocyanin (ACN) and non-anthocyanin polyphenol (PP) fraction on the intracellular ROS production (**A**,**B**) and antioxidant and pro-oxidant enzymes mRNA expression (**C**,**D**) in hypertrophied 3T3-L1 adipocytes. Data are the mean values ± SD (*n* = 3). ^a^ *p* < 0.05, ^b^ *p* < 0.01, ^c^ *p* < 0.001.

**Figure 2:**
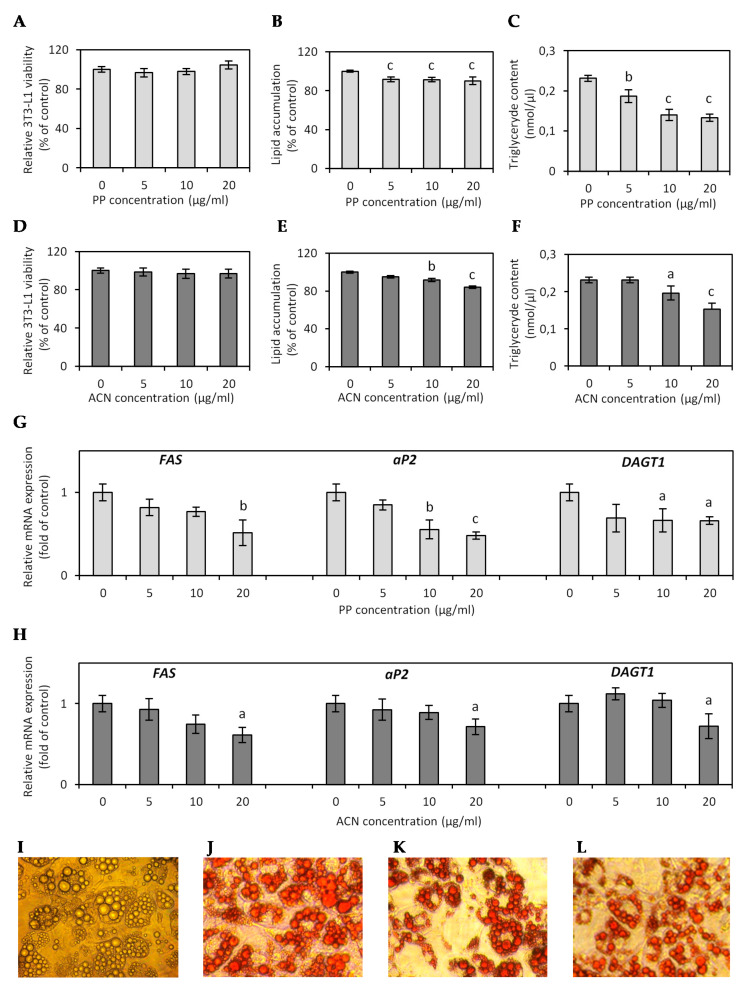
Effect of non-anthocyanin polyphenol (PP) fraction and anthocyanin (ACN) fraction on cell viability (**A**,**D**), lipid accumulation (**B**,**E**), triglyceride content (**C**,**F**), and lipogenic gene expression (**G**,**H**) in hypertrophied 3T3-L1 adipocytes. Data are the mean values ± SD (*n* = 3).^a^ *p* < 0.05, ^b^ *p* < 0.01, ^c^ *p* < 0.001. The photos present hypertrophied 3T3-L1 adipocytes on day 12 after differentiation (**I**), Oil Red-stained hypertrophied 3T3-L1 adipocytes non-treated (**J**), and treated with PP fraction (**K**) and ACN fraction (**L**) at the concentrations of 20 μg/mL. The cells were photographed at a magnification of 100×.

An animal study has shown that obesity is characterized by increased vascular oxidative stress and endothelial dysfunction [15]. Enzymatic sources contributing to increased ROS production in pathophysiological states such as obesity are xanthine oxidase, NADH/NADPH oxidase, and inducible nitric oxide synthase (iNOS) [16]. Oxidative stress contributes to endothelium dysfunction via inactivation of nitric oxide (NO) by superoxide and other ROS; thus, diet intervention rich in antioxidants which prevent their production might ultimately correct endothelial dysfunction [16]. Therefore, the potential of ACN and PP fractions derived from lingonberry fruit to mitigate oxidative stress in hypertrophied adipocytes was evaluated. The results obtained in the NBT assay indicate that the PP fraction decreased ROS accumulation in adipocytes in a dose-dependent manner (Figure 1A). The PP fraction at concentrations of 5, 10, and 20 µg/mL reduced the ROS production by 10.5%, 12.1%, and 15%, respectively (*p* < 0.01). In contrast, the ACN fraction did not significantly influence intracellular ROS production (Figure 1B). Moreover, it should be noted that both PP and ACN fraction did not affect adipocyte viability (Figure 2A), indicating that the PP inhibitory effect on intracellular ROS generation was not due to cytotoxicity. NADPH oxidase 4 (NOX4) from NOX family NADPH oxidases is considered the primary ROS synthesis source in adipose tissue [16]. The NOX-enhanced ROS generation in hypertrophied adipocytes decreased the production of the insulin-sensitizing, antiatherogenic and anti-inflammatory factors. It decreased the mRNA expression of antioxidant defense enzymes, including superoxide dismutase (SOD), catalase, and glutathione peroxidase (GPx) [17]. Furthermore, iNOS, an inducible pro-inflammatory enzyme, is overexpressed in obese adipose tissue, and disruption of the iNOS gene protected obese mice from insulin resistance development [18]. The oxidant imbalance in obese patients causes endothelial dysfunction and leads to increased blood pressure and coronary artery disease [19]. Our previous study has shown that lingonberry fruit extract reduces ROS generation in inflamed adipocytes by increasing the expression of antioxidant enzymes (SOD2, catalase, GPx) and decreasing a pro-oxidant enzyme (NOX4) [11]. In the current study, the antioxidant effect of lingonberry-derived ACN and PP fractions was evaluated in hypertrophied 3T3-L1 adipocytes. Real-time PCR analysis showed that the PP fraction at the highest dose of 20 µg/mL significantly downregulated *NOX4* (↓40%, *p* < 0.01) and *iNOS* (↓37%, *p* < 0.05), and upregulated *SOD2* (↑82%, *p* < 0.001) mRNA expression (Figure 1C). The ACN fraction at a dose of 20 µg/mL inhibited *NOX4* by 33% (*p* < 0.01), *iNOS* by 37% (*p* < 0.01), and enhanced the expression of *SOD2* by 23% (*p* > 0.05) (Figure 1D). The obtained results indicate that the lingonberry fruit antioxidant potential is probably associated with upregulation of *SOD2* expression and downregulation of *iNOS* expression by the PP fraction and ACN fraction. In addition, the compounds from both PP and ACN fractions were found to inhibit *NOX4* expression.

Yen et al. (2011) investigated the effect of 21 polyphenolic compounds on oxidative stress in 3T3-L1 adipocytes induced by TNF-α. They found that that *p*-coumaric acid, quercetin, and resveratrol enhance antioxidant defense enzymes, including SOD2, GPx, glutathione, and glutathione S-transferase [20]. Cyanidin-3-glucoside, an anthocyanin derivative commonly found in different berries, reduced the intracellular ROS production in adipocytes induced by H_2_O_2_ or TNF-α [21].

Polyphenol-rich plant extracts significantly reduced ROS generation induced in 3T3-L1 cells by H_2_O_2_, and this effect was associated with an increase in SOD2 gene expression [22]. Animals fed a diet inducing oxidative stress and supplemented with lingonberry extract (23 mg/kg of body weight) had a decreased total oxidant status by 25%, and increased levels of antioxidant enzymes: SOD, catalase, and glutathione reductase in red blood cells and liver [23].

### 3.3. Effect of ACN and PP Fractions on Lipid Accumulation in Hypertrophied 3T3-L1 Adipocytes

**Figure 3:**
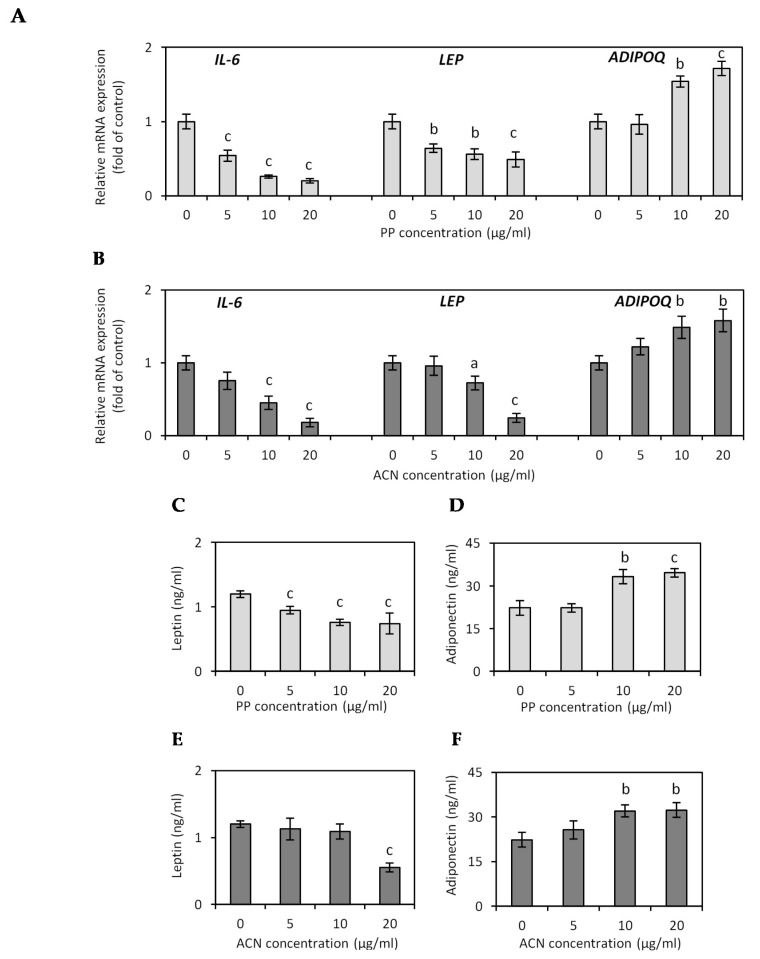
Effect of non-anthocyanin polyphenol (PP) and anthocyanin (ACN) fractions on adiponectin (ADIPOQ), leptin (LEP), and interleukin-6 (IL-6) gene expression (**A**,**B**), and adiponectin (**D**,**F**) and leptin (**C**,**E**) protein secretion by hypertrophied 3T3-L1 adipocytes. Data are the mean values ± SD (*n* = 3). ^a^ *p* < 0.05, ^b^ *p* < 0.01, ^c^ *p* < 0.001.

In this study, hypertrophic 3T3-L1 adipocytes, formed following the prolonged cultivation of differentiated mature adipocytes in high glucose conditions with medium replacement in 2-day intervals, displayed a morphological pattern typical for adipocyte hypertrophy with disturbance of the lipid handling processes. The 3T3-L1 adipocytes reached the critical cell size and became lipid-overloaded, largely occupied by fat droplets, as shown in Figure 2I. The effect of ACN and PP fractions on lipid content in the hypertrophied 3T3-L1 adipocytes was determined by Oil Red *O* staining and measurement of the total TG concentration on the cellular level. Semi-quantitative Oil Red *O* staining revealed that the PP fraction at concentrations of 5, 10, and 20 µg/mL reduced lipid accumulation by 4.9%, 8.4%, and 16% (*p* < 0.001) compared to untreated cells (Figure 2B,J,K), while the ACN fraction at the same concentrations reduced lipid content by 8.4%, 8.6% (*p* < 0.01), and 9.8% (*p* < 0.001), respectively (Figure 2E,J,L). Quantitative analysis of TG content in the cells have shown that 24-h treatment of hypertrophied adipocytes with the PP fraction decreased the TG content by 19.4% (*p* < 0.01), 49.6%, and 42.4% (*p* < 0.001) at a concentration of 5, 10, and 20 µg/mL, respectively (Figure 2C). The effect of the ACN fraction on TG content was less profound, and only the highest dose of ACN fraction 20 µg/mL decreased lipid accumulation by 9.8% (*p* < 0.001), and TG content by 33.9% (*p* < 0.001) (Figure 3F).

The effect of both fractions on lipid accumulation was confirmed by real-time PCR analysis of the expression of genes *FAS* (*fatty acid synthase*), *DGAT1* (*diacylglycerol acyltransferase 1*), and *aP2* (*fatty acid-binding protein*) involved in fatty acid (FA) and TG synthesis. Animal models with genetic modifications have shown that adipogenic and lipogenic genes, including *FAS*, *DGAT1*, and *aP2,* play a fundamental role in FA and TG synthesis and lipid storage, and a high-fat (HF) diet significantly increased the relative expression of these genes in adipose tissue [24]. DGAT1 is highly expressed in adipose tissue and catalyzes the final reaction of TG synthesis. DGAT1-deficient animals are resistant to obesity and more sensitive to insulin and leptin; therefore, inhibition of DGAT1 may be a potential strategy for decreasing TG synthesis for treating obesity [25]. Fatty acid-binding protein 4 (FABP4), also named adipocyte FABP or aP2, is mostly expressed in fat cells and plays significant roles in developing insulin resistance and atherosclerosis concerning metabolically driven low-grade and chronic inflammation [26]. Circulating aP2 levels are associated with several aspects of metabolic syndrome and endothelial dysfunction; thus, inhibition of the aP2 function could be a novel therapeutic strategy for several diseases, including obesity and cardiovascular disease [27]. FAS is also highly expressed in adipose tissue, and enhanced FAS expression correlates to visceral fat accumulation, impaired insulin sensitivity, and intensified pro-inflammatory cytokine production [28].

In our study, real-time PCR analysis showed that the PP and ACN fraction treatment dose-dependently inhibited *DAGT1*, *aP2*, and *FAS* mRNA expression (Figure 2G,H), but the PP effect was more significant. The PP fraction decreased expression of *DAGT1* in the range of 31–34% (*p* < 0.05). The effect of the PP fraction with statistical significance on *aP2* and *FAS* expression was observed only at concentrations of 10 and 20 µg/mL with 44.7% (*p* < 0.01) and 51.9% (*p* < 0.001) decreases in *aP2* expression and with 23.3% and 48.6% (*p* < 0.01) decreases in *FAS* expression (Figure 2G). The ACN fraction only at the highest dose of 20 µg/mL downregulated *FAS, aP2*, and *DAGT1* expression by approximately 28% (*p* < 0.05) (Figure 2H).

In vivo study has shown that DGAT1-deficient mice (*Dgat1^−/−^*) had less adipose mass and smaller adipocytes. Despite reduced tissue TG levels, the diacylglycerol and fatty acyl CoA, substrates of the DGAT reaction, were not significantly elevated in skeletal muscle and liver. Moreover, the serum TG level was normal in *Dgat1^−/−^* mice [25]. DGAT1 deficiency also altered the endocrine function of WAT. Adiponectin mRNA expression in WAT was increased 2-fold in *Dgat1^−/−^* mice fed an HF diet [29]. The aP2-deficient mouse model revealed a slight increase in plasma FA. An elevated FA was found to link with the development of obesity and insulin resistance, but paradoxically, mice lacking aP2 were more sensitive to insulin [30].

Many natural products from plants have been identified as potent DGAT inhibitors [31]. Rose petals, rich in polyphenols and free gallic acid had high antioxidant activity and the ability to inhibit TG synthesis. An extract of rose petals showed selective DGAT inhibition without suppressing other microsomal enzymes [32]. Anthocyanin-rich extracts effectively decreased body weight gain and accumulation of lipids by decreasing the mRNA level and inhibiting FA and TG synthesis enzymes and lipogenic activity [33]. Heyman et al. (2014) have found that lingonberries prevented adiposity, hepatic lipid accumulation, and dyslipidemia in mice fed an HF diet [34]. Qin et al. (2011) have found that consumption of the chokeberry extract, rich in polyphenols, reduces weight gain and epididymal fat accumulation; at the molecular level, it inhibits *aP2*, *FAS,* and *LPL* mRNA expression [35].

### 3.4. Effect of ACN and PP Fractions on Adipokine and Inflammatory Cytokine Expression in Hypertrophied 3T3-L1 Adipocytes

Adiponectin is the most abundant peptide secreted by adipocytes. Adiponectin production, which has a beneficial effect on insulin sensitivity and cardiovascular function, is significantly reduced in obese adipose tissue. Numerous epidemiological studies have shown that adiponectin deficiency is an independent risk factor for endothelial dysfunction [36]. Adiponectin exerts protective effects by inhibiting TNF-α, resistin, and adhesion molecules (VCAM-1, ICAM-1, and E-selectin) in endothelium and increasing endothelial NO production [37]. The most available therapy for cardiovascular diseases is lifestyle modifications by calorie restriction and dietary interventions that increase plasma levels of adiponectin. There is also a growing interest in the pharmaceutical industry to search for natural compounds that can increase adiponectin production [36].

**Figure 4:**
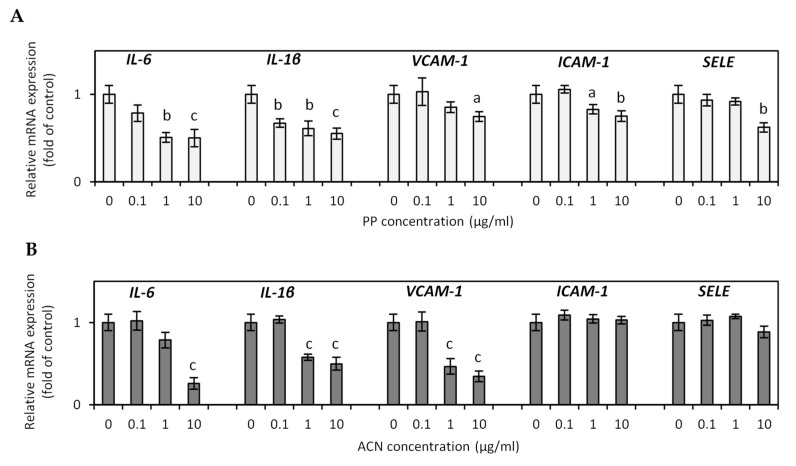
Effect of non-anthocyanin polyphenol (PP) fraction (**A**) and anthocyanin (ACN) fraction (**B**) on inflammatory-related gene expression in HUVECs. Data are the mean values ± SD (*n* = 3). ^a^ *p* < 0.05, ^b^ *p* < 0.01, ^c^ *p* < 0.001.

In our study, adiponectin mRNA expression in hypertrophied adipocytes after PP treatment was upregulated, with a significant enhancement by 57.0% (*p* < 0.01) and 72.7% (*p* < 0.001) at the concentrations of 10 and 20 µg/mL (Figure 3A). The ACN fraction at concentrations of 10 and 20 µg/mL increased adiponectin expression by 50.5% and 59.6%, respectively (*p* < 0.01) (Figure 3B). Contrary to adiponectin, the serum level of leptin is elevated in obesity due to increased leptin release from large hypertrophic adipocytes compared with small fat cells [38]. Evidence from clinical trials and animal experiments suggests that hyperleptinemia is involved in the pathogenesis of obesity-related cardiovascular disease and endothelial dysfunction due to ROS-mediated NO inactivation [39]. In vitro studies have shown that leptin increases ROS production in endothelial cells [40]. In this research, a significant reduction in leptin expression was found after treatment of adipocytes with ACN and PP fractions (Figure 3A,B). Reduction of leptin expression by 27.3% and 75.8% was obtained in hypertrophied adipocytes treated with the ACN fraction at the concentrations of 10 (*p* < 0.05) and 20 µg/mL (*p* < 0.001) (Figure 3B). The PP fraction decreased leptin mRNA expression by 35.0%, 43.4%, and 50.5% at a concentration of 5, 10 (*p* < 0.01), and 20 µg/mL (*p* < 0.001), respectively (Figure 3A). A similar effect of PP and ACN fractions was observed on leptin (Figure 3C,E) and adiponectin (Figure 3D,F) secretion. Treatment of hypertrophied adipocytes with the PP fraction increased the secretion of adiponectin by 49.3% and 55.2% at a concentration of 10 (*p* < 0.01) and 20 µg/mL (*p* < 0.001), respectively (Figure 3D). All tested concentrations of PP fraction (5, 10, and 20 µg/mL) decreased leptin secretion (↓20.8%, ↓36.7%, and ↓38.1%; *p* < 0.001) (Figure 3C). Only at a concentration of 20 µg/mL did the ACN fraction significantly decrease leptin secretion (↓53.9%, *p* < 0.001) (Figure 4E). At doses of 10 and 20 µg/mL, the ACN fraction increased adiponectin production by 43.3% and 44.8% (*p* < 0.01), respectively (Figure 3F).

Moreover, adipocytokines such as IL-1 and IL-6 are closely linked to endothelial dysfunction and subclinical inflammation [41]. Yudkin et al. (2002) have shown, in healthy subjects, relationships between levels of a hepatic acute-phase C-reactive protein (CRP) and levels of IL-6 released from obese adipose tissue, indicating adipose tissue as a major source for circulating IL-6 [41]. The study with 368 participants showed that persistently high levels of IL-6 were associated with a higher body mass index and an increased number of cardiovascular diseases compared to persistently lower levels of IL-6 [42]. Therefore, we investigated the effect of ACN and PP fractions on the expression of IL-6 in hypertrophied adipocytes after 24-h treatment. Compared to the control adipocytes, the PP fraction downregulated the expression of IL-6 by 45.5%, 73.7%, and 79.8% at a dose of 5, 10, and 20 µg/mL (*p <* 0.001) (Figure 3A). The ACN fraction suppressed IL-6 mRNA expression by 54.5% and 82.0% at a concentration of 10 and 20 µg/mL (*p <* 0.001), compared to untreated adipocytes (Figure 3B).

Our previous study showed that lingonberry fruit extract suppressed pro-inflammatory cytokines IL-6, IL-1β, and leptin expression, and significantly enhanced the expression of anti-inflammatory cytokines IL-10 and adiponectin in TNF-α-induced 3T3-L1 cells [11]. In mice fed the HF diet and supplemented with chokeberry juice concentrate, a higher plasma adiponectin level was observed [43]. Qin et al. (2012) have found that chokeberry extract elevated plasma adiponectin and inhibited plasma TNF-α and IL-6 levels in rats fed a high-fructose diet [35]. C57BL/6J mice fed the HF diet had elevated serum levels of TG, cholesterol, and leptin. Purified ACNs provided along with the HF diet led to decreasing serum TG, cholesterol, and leptin to the low-fat diet levels [44]. Tsuda et al. (2004) also found that adiponectin gene expression was upregulated in the WAT of ACN-fed mice [45].

Twenty healthy volunteers supplemented for 4 weeks with 200 mL/day of ACN-rich Queen Garnet plum juice for 4 weeks had a significantly reduced body weight and BMI with an average decrease of 0.6 kg in body weight and 0.2 units in BMI. Furthermore, consumption of ACN-rich plum juice significantly increased adiponectin blood levels (average increase of 3.8 µg/mL) and decreased leptin blood levels (average decrease of 1.3 ng/mL) [46]. A study conducted by Vugic et al. (2020) showed that the regular intake of ACNs reduced obesity-associated inflammation in obese subjects. The supplementation with purified ACNs for 28 days significantly reduced the plasma IL-6 level [47]. Several in vitro and in vivo studies confirmed that ACN-rich food consumption prevents obesity-related consequences such as diabetes, inflammation and oxidative stress. ACN supplementation favorably alters genes involved in glucose, FA and lipid metabolism, immune and inflammatory system, antioxidant defense, and the antiangiogenic system [48].

### 3.5. The Effects of PP and ACN Fractions on TNF-α-Induced Endothelial Dysfunction

The endothelium plays a vital role in vascular homeostasis and response to various stimuli, synthesizing and releasing many vasoactive substances, growth modulators, and other elements that mediate/influence these functions. The loss of balance between pro-atherogenic and antiatherogenic factors production leads to endothelial dysfunction [49]. The plasma levels of markers of endothelial activation, such as vascular cell adhesion molecule (VCAM), intercellular adhesion molecule (ICAM), endothelin 1 (ET-1), E-selectin (SELE), and markers of low-grade inflammation such as CRP, IL-1β, and IL-6 indicate the endothelial dysfunction [7]. Obesity has been confirmed to activate endothelial cell functions. It has been shown that endothelial cells of obese mice express higher levels of ICAM-1 [50].

Our study examined the ability of ACN and PP fractions to decrease endothelial dysfunction in HUVECs induced by TNF-α. Accumulating evidence from clinical trials and basic research proves a crucial role of TNF-α in vascular dysfunction and vascular disease [51]. TNF-α is a proinflammatory cytokine with multiple immune response functions, playing a pivotal role in low-grade systemic inflammation. TNF-α-mediated signaling pathways initiate and stimulate atherosclerosis, thrombosis, vasculitis, vascular oxidative stress, and endothelial cell apoptosis, contributing to vascular impairment [51]. A close relationship between TNF-α upregulation and lipid metabolism and HF and high-carbohydrate diets has been reported in several studies. Significantly increased plasma levels of TNF-α, IL-6, ICAM-1, and VCAM-1 have been observed in patients with hyperlipidaemia, obesity, metabolic syndrome, and type 2 diabetes [51,52]. In our study, TNF-α significantly stimulated several inflammation-related genes and adhesion molecules such as IL-6, IL-1β, VCAM-1, ICAM-1, and SELE in HUVECs. Compared to control cells, PP and ACN fractions decreased TNF-α-induced increase in IL-6, IL-1β, VACM-1, and ICAM-1 expression in a dose-dependent manner (Figure 4A,B). After incubation of HUVECs with a PP fraction at a concentration of 10 µg/mL, *IL-6* and *IL-1β* mRNA expression decreased by 49.6% and 45.0% (*p* < 0.001), respectively. The mRNA expression of *VCAM-1*, *ICAM-1*, and *SELE* decreased by 25.5% (*p* < 0.05), 25.0% (*p* < 0.01), and 38.0% (*p* < 0.01) (Figure 4A). The ACN fraction at a dose of 10 µg/mL suppressed *IL-6*, *IL-1β*, and *VCAM-1* mRNA expression by 74.0%, 50.0%, and 65.6%, respectively (*p* < 0.001). The ACN fraction did not affect *ICAM-1* and *SELE* expression (Figure 4B). In TNF-α-induced HUVECs, the upregulated VCAM-1, ICAM-1, and E-selectin were meaningfully reduced by pretreatment with quercetin [53]. ACNs and hydroxycinnamic acids present in blueberry and cranberry fruits reduced TNF-α-induced upregulation of various inflammatory mediators (IL-8, MCP-1, and ICAM-1) in HUVECs [54]. A clinical study with 27 subjects with metabolic syndrome has shown that consumption of freeze-dried strawberry for 8 weeks decreased circulating levels of VCAM-1 by 18%, while no effects were noted in ICAM-1 [55]. Ruel et al. (2008) reported a significant decrease in adhesion molecules (ICAM-1 and VCAM-1) in healthy volunteers after a 12-week supplementation with low-calorie cranberry juice [56]. Mechanisms that link obesity and endothelial dysfunctions are multidirectional and complex. Several clinical studies have shown that reducing WAT hypertrophy leads to decreased plasma levels of various adipocytokines, attenuates the pro-inflammatory state, and improves endothelial functions [57].

In summary, the results have shown that PP and ACN fractions obtained from lingonberry fruit ameliorate adipocyte hypertrophy by acting directly on the molecular and cellular pathways. Both fractions decreased intracellular ROS generation by enhancing the expression of antioxidant defense enzyme SOD2 and inhibiting oxidant enzymes such as NOX4 and iNOS. Moreover, PP and ACN fractions downregulated the expression of *FAS*, *DGAT1*, and *aP2*, which resulted in reduced TG content in adipocytes. Both fractions downregulated the expression of pro-inflammatory mediators (IL-6 and leptin), and upregulated adiponectin expression. To our knowledge, the present study is the first to show the protective effect of PP and ACN fractions from lingonberry fruit on endothelial functions by significantly decreasing the expression of several inflammation-related genes and adhesion molecules such as *IL-6*, *IL-1β*, *VCAM-1*, *ICAM-1* and *SELE* in TNF-α-induced HUVECs. These results suggest that consuming polyphenol-rich lingonberry fruit may help prevent and treat obesity and endothelial dysfunction due to their antioxidant and anti-inflammatory actions. Thus, lingonberries could be a dietary recommendation for preventing and managing obesity and cardiovascular complications, although further in vivo studies in animal models, followed by clinical trials, are needed.

## Figures and Tables

**Table 1:** Compounds identified in anthocyanin (ACN) fraction (**A**) and non-anthocyanin polyphenol (PP) fraction (**B**) obtained from lingonberry fruit.

**(A)**					
**Compound**	**RT**	**Precursor Ion (m/z)**	**Ionization Mode**	**Product Ion (m/z)**	**Contribution**
	**(min)**				**(%)**
Cyanidin-3-*O*-galactoside	16.25	449.1126	(+)	287.0611	82.5
Cyanidin-3-*O*-glucoside	17.17	449.1124	(+)	287.0589	4.5
Cyanidin-3-*O*-arabinoside	18.24	419.1021	(+)	287.0607	13
**(B)**					
**Compound**	**RT**	**Precursor Ion (m/z)**	**Ionization Mode**	**Product Ion (m/z)**	**Contribution**
	**(min)**				**(%)**
**Flavan-3-ols**					40.4
B-type procyanidin dimer	8.46	577.134	(−)	407.0763	6.3
(+)/(−)-Catechin	10.49	289.1139	(−)	245.1203	8.3
(+)/(−)-Epicatechin	12.42	289.114	(−)	245.121	4.2
B-type procyanidin dimer	13.16	577.1338	(−)	407.0762	1.6
B-type procyanidin dimer	13.74	577.1349	(−)	407.0769	3.1
A-type procyanidin dimer	19.36	575.1195	(−)	449.0887	6.2
A-type procyanidin trimer	22.31	863.1829	(−)	575.1217	10.7
**Hydroxycinnamic acid derivatives**					**22.8**
3-*O*-Caffeoylquinic acid	12.42	353.134	(−)	191.0907	8.1
Ferulic acid−hexoside	13.71	355.1037	(−)	193.05	2.9
Ferulic acid−hexoside	14.14	355.1041	(−)	193.0503	2.6
2′-*O*-Caffeoylarbutin	19.36	433.1137	(−)	179.0337	5
Ferulic acid−hexoside	21.7	355.1031	(−)	193.0509	2.5
Coumaroyl-hexose−hydroxyphenol	22.25	417.1065	(−)	163.0396	1.8
**Flavonols**					**31**
Quercetin-3-*O*-galactoside	23.46	463.0874	(−)	301.0334	7.8
Quercetin-3-*O*-glucoside	24.17	463.0882	(−)	301.0354	1.3
Quercetin-3-*O*-xyloside	24.85	433.0777	(−)	301.0336	1.9
Quercetin-3-*O*-arabinofuranoside	27.05	433.0776	(−)	301.0327	6.3
Quercetin-3-*O*-rhamnoside	27.87	447.0935	(−)	301.0349	8.1
Quercetin	34.37	301.0356	(−)	151.0031	5.6
**Anthocyanins**					**5.8**
Cyanidin-3-*O*-galactoside	16.28	449.1126	(+)	287.0611	2.6
Cyanidin-pentoside	21.33	419.1033	(+)	287.0618	2
Cyanidin 3-*O*-(6″-acetyl)-glucoside	23.82	491.1507	(+)	287.0621	1.2

## Supplementary Materials

The following are available online at https://www.mdpi.com/2072-6643/13/3/885/s1, Table S1: The primers sequence used for real-time PCR.
