# Supplementary material for: ROS Modulating Effects of Lingonberry (Vaccinium vitis-idaea L.) Polyphenols on Obese Adipocyte Hypertrophy and Vascular Endothelial Dysfunction
Source: Nutrients. 2021 Mar 9;13(3):885. doi: 10.3390/nu13030885 (PMC7999824; doi:10.3390/nu13030885)
Supplement: Supplementary file 1 [file nutrients-13-00885-s001.pdf]

**Table S1.** The primers sequence used for real-time PCR

| Gene | Accession | No.Sequence (5' – 3') | Amplicon (bp) |
|---|---|---|---|
| **Mm *IL-6*** | NM-031168.1 | F:TCTGAAGGACTCTGGCTTTG<br>R:GATGGATGCTACCAAACTGGA | 142 |
| **Mm *NOX4*** | NM-015760.5 | F:GATCACAGAAGGTCCCTAGCAG<br>R:GTTGAGGGCATTCACCAAGT | 134 |
| **Mm *LEP*** | NM-008493 | F:GGATCAGGTTTTGTGGTGCT<br>R:TTGTGGCCCATAAAGTCCTC | 187 |
| **Mm *SOD2*** | NM-013671.3 | F:CGTGTCTGTGGGAGTCCAAGGTTCAG<br>R:GTCAATCCCCAGCAGCGGAATAAG | 139 |
| **Mm *ADIPOQ*** | NM-009605 | F:CTGGCCACTTTCTCCTCATTTC<br>R:GGCATGACTGGGCAGGATTA | 120 |
| **Mm *FABP4*** | NM-024406 | F:TCACCTGGAAGACAGCTCCT<br>R: AATCCCCATTTACGCTGATG | 182 |
| **Mm *DAGT1*** | NM-010046.3 | F: TTCCGCCTCTGGGCATT<br>R: AGAATCGGCCCACAATCCA | 67 |
| **Mm *iNOS*** | NM-010927.3 | F: TGA AGA AAA CCC CTT GTG CT<br>R: TTCTGTGCTGTCCCAGTGAG | 100 |
| **Mm *FAS*** | NM-007988 | F: TTGCTGGCACTACAGAATGC<br>R:AACAGCCTCAGAGCGACAAT | 192 |
| **Mm *ACTB*** | NM-007393 | F:CCA CAG CTG AGA GGG AAA TC<br>R:AAG GAA GGC TGG AAA AGA G | 193 |
| **Hs *IL-6*** | NM-001371096.1 | F:GCAGAAAACAACCTGAACCTT<br>R:ACCTCAAACTCCAAAAGACCA | 116 |
| **Hs *VCAM1*** | NM-001078.4 | F:CAGGCTAAGTTACATATTGATGACAT<br>R:GAGGAAGGGCTGACCAAGAC | 116 |
| **Hs *ICAM1*** | NM-000201.3 | F:GTATGAACTGAGCAATGTGCAAG<br>R:GTTCCACCCGTTCTGGAGTC | 119 |
| **Hs *SELE*** | NM-000450.2 | F: AGAGTGGAGCCTGGTCTTACA<br>R: CCTTTGCTGACAATAAGCACTGG | 77 |
| **Hs *IL-1β*** | NM-000576.3 | F:CCTGAAGCCCTTGCTGTAGT<br>R:AGCTGATGGCCCTAAACAGA | 112 |
| **Hs *GAPDH*** | NM 002046.7 | F: AATGAAGGGGTCATTGATGG<br>R: AAGGTGAAGGTCGGAGTCAA | 108 |
